# Streptococcus viridans Liver Abscess and Septicemia Likely Secondary to a Swallowed Dental Bridge

**DOI:** 10.7759/cureus.49998

**Published:** 2023-12-05

**Authors:** Shannon Monro, Eleonora Feketeova

**Affiliations:** 1 Internal Medicine, Garnet Health Medical Center, Middletown, USA; 2 Medical Education, Garnet Health Medical Center, Middletown, USA

**Keywords:** streptococcus viridans liver abscess, septicemia, streptococcus viridans, foreign body, liver abscess

## Abstract

Pyogenic liver abscesses are relatively rare in developed countries such as the United States, and, when they do occur, they are typically secondary to direct or hematogenous spread of intra-abdominal pathologies. Gastrointestinal pathogens such as *Escherichia coli* and *Enterococcus* species are typically implicated. Conversely, the *Streptococcus viridans* group is a rare cause of bacteremia and abscess formation, especially in immunocompetent patients. We present a case of a 53-year-old male who presented with *S. viridans* liver abscess that was found to be secondary to a swallowed dental bridge that was lodged in the patient’s descending colon. The patient was treated with intravenous antibiotics, percutaneous drainage, and colonoscopy for removal of the foreign body; the patient had a good response to treatment and was discharged on oral antibiotics. In any patient who has fever and abnormal liver function tests, hepatobiliary sepsis including liver abscess should always be excluded. Additionally, it is important to suspect unusual pathogens and sources of infection. We suggest empiric broad-spectrum antibiotic coverage when liver abscess is suspected and tailoring treatment as the specific organism and susceptibilities are identified. Moreover, we suggest the importance of removing any foreign bodies promptly upon discovery as they may serve as an important nidus of infection.

## Introduction

Pyogenic liver abscesses (PLA) are relatively rare in developed countries such as the United States, with an incidence of approximately 2.3-3.7 per 100,000 [1]. When they do occur, they are usually caused by direct or hematogenous spread from gastrointestinal pathologies such as gallbladder or appendiceal disease [2] and are more common in immunocompromised patients. The majority of causal pathogens are *Escherichia coli* and other *Enterococcus* species [2]. Conversely, the viridans group streptococci are rarely implicated in diseases such as PLA [2] and are commensal organisms of the oral cavity, most commonly causing dental disease. *Streptococcus viridans*, however, do have the ability to form biofilms, which can aid in antibiotic resistance and immune system evasion [3], especially in the presence of the proper substrate, such as a foreign body. 

In this case report, we report an interesting case of a large pyogenic liver abscess because of *S. viridans* secondary to a swallowed dental bridge.

## Case presentation

A 53-year-old male with a past medical history of schizoaffective disorder presented to the emergency department (ED) of a community medical center by emergency medical services (EMS) for confusion. In the ED, the patient was found to be a poor historian and was noncompliant with his medications for schizoaffective disorder. Social history was significant for occasional alcohol/drug use. The patient denied any recent travel. He had a normal heart rate, respiratory rate, and oxygen saturation. Physical examination was benign. Labs were significant for leukocytosis of 15.8 WBC/uL (reference range: 4.3-10.6 x 10^3^ microliters), transaminitis with an AST of 113 U/L (reference range: 15-41 U/L), and ALT of 86 U/L (reference range 17-63 U/L) and elevated alkaline phosphatase of 222 U/L (reference range: 45-117 U/L). A chest x-ray was significant for infiltrate in the right lower lobe consistent with atelectasis or early infiltrate. The patient was admitted to the community medical center for aspiration pneumonia and was empirically treated with intravenous ceftriaxone 1 g once daily and intravenous azithromycin 500 mg once daily. 

**Figure 1:**
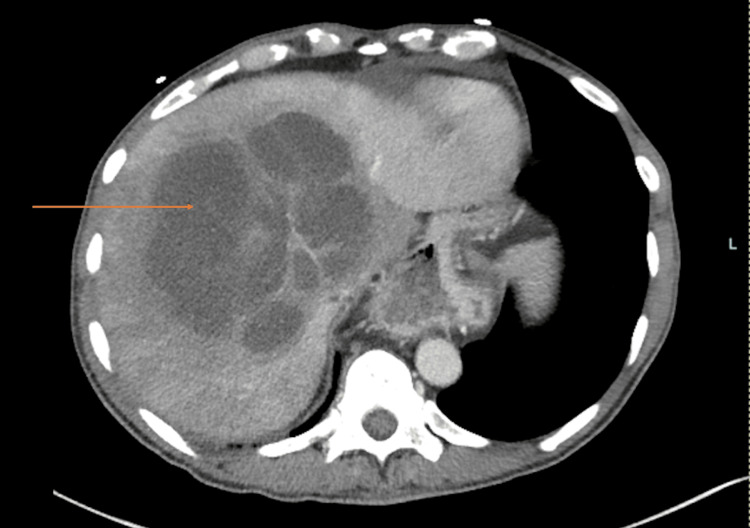
Axial CT scan of the patient's chest, abdomen, and pelvis taken on admission, showing a liver abscess measuring approximately 13 by 12 by 12 cm (indicated by the arrow).

**Figure 2:**
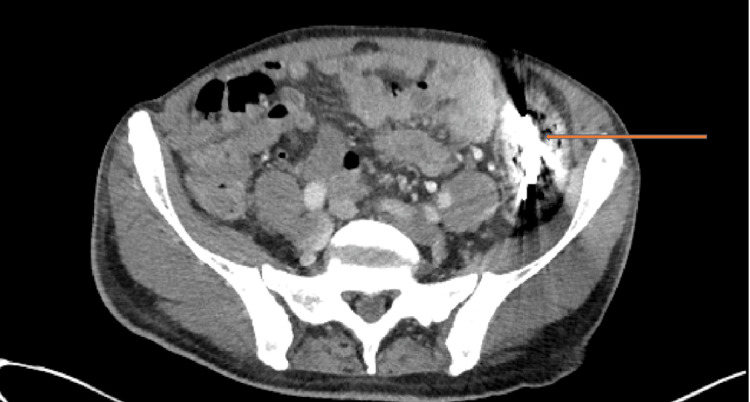
Axial CT scan of the patient's chest, abdomen, and pelvis taken on admission, showing a metallic foreign body of unknown origin in the descending colon, indicated by the arrow.

Upon admission, the patient was febrile to 101 degrees Fahrenheit. Because of the patient’s elevated liver function tests, imaging was ordered. Ultrasound of the liver was significant for a liver mass. Furthermore, a computed tomography (CT) scan of the chest, abdomen, and pelvis revealed a large, multiloculated liver mass measuring approximately 13 by 12 by 12 centimeters and a metallic foreign body in the patient’s descending colon (Figures 1-2). The patient's antibiotics were switched to piperacillin-tazobactam 3.375 g four times daily for broad-spectrum coverage. Furthermore, the patient was arranged to undergo image-guided abscess drainage by interventional radiology. Following the drainage of his liver abscess, the patient made little clinical progress and developed paleness and rigor, indicating worsening infection and possible septicemia.

Blood cultures taken on admission grew *S. viridans*, and vancomycin 1250 milligrams twice daily was added to the patient’s treatment regimen. *Entamoeba histolytica* antibody, HIV/hepatitis tests, *Echinococcus* antibody, alpha-fetoprotein (AFP) assay, and carcinoembryonic antigen (CEA) were all negative. Following treatment with vancomycin for four days, the patient’s blood cultures were negative; however, serial imaging revealed the patient’s liver abscess had not resolved following image-guided drainage. Thus, interventional radiology was reconsulted for repositioning of the drain. Because of diminishing clinical response, the patient’s antibiotic regimen was again changed, and he received ertapenem 1 g once daily for the remainder of his hospital stay.

**Figure 3:**
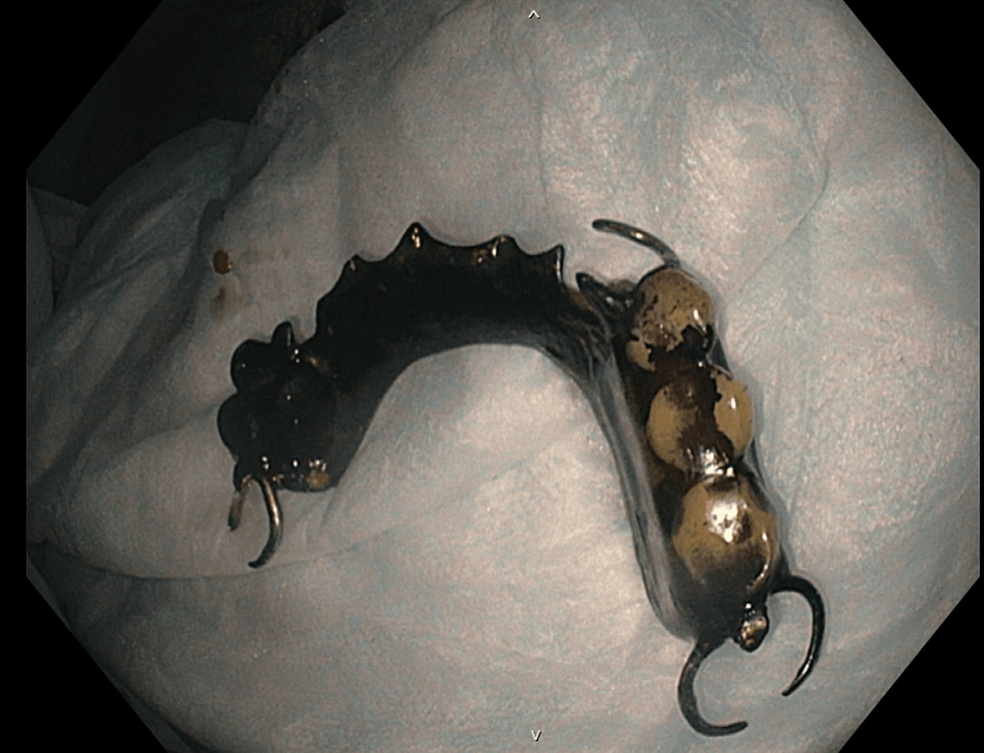
Image of the foreign body, found to be a swallowed dental bridge, status-post removal by colonoscopy.

**Figure 4:**
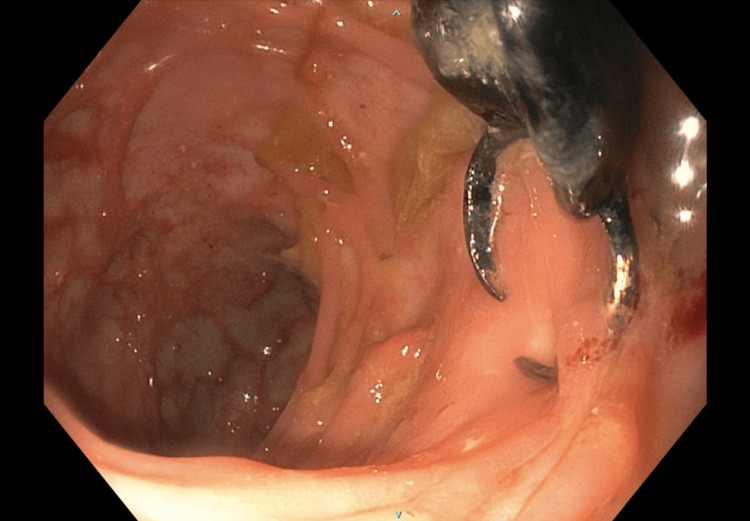
Colonoscopy images showing swallowed dental bridge piercing descending colonic diverticula.

Additionally, it was determined that the foreign body was a potential source of infection and that the object should be removed as the patient was not improving after intravenous antibiotics and percutaneous drainage. The patient underwent two courses of bowel preparation in an attempt to flush the foreign body; however, the patient was unable to pass the object. The patient subsequently underwent a colonoscopy for retrieval of the foreign body which was found to be a swallowed dental bridge (Figure 3). The dental bridge’s barbs punctured the patient’s diverticula and caused it to become lodged in the descending colon (Figure 4). The patient was also found to have a large right-sided pleural effusion that was drained. 

Repeat CT imaging done one month from admission showed significant improvement of the abscess, with a residual size of 5.2 by 2.8 cm, along with focal inflammation of the colon wall where the foreign body was previously located. Ultimately, the patient was discharged on oral amoxicillin-clavulanate 500 mg twice daily for four weeks and was to be monitored outpatient with weekly lab work and biweekly imaging to assess further clinical response.

## Discussion

PLA are a serious and potentially life-threatening condition that involves the formation of a pus-filled cavity in the liver parenchyma. These abscesses are rarely seen in developed countries, but when they do occur, they are usually secondary to direct or hematogenous spread of some kind of biliary or gastrointestinal pathology [4]. They are also more common in immunocompromised hosts, with conditions that predispose patients to form PLA being neoplasm, diabetes, and other autoimmune diseases, and they are rarely seen in patients without these comorbid conditions [5]. The most common abscess-causing pathogens are *E. coli *and *Klebsiella pneumoniae* [4]. Management typically involves multidisciplinary treatment with antibiotics and percutaneous or surgical drainage. If left undertreated or untreated, PLA can result in peritonitis, sepsis, shock, and death.

*S. viridans* is a group of gram-positive, catalase, and coagulase-negative facultative anaerobes that are a commensal organism mainly found in the oral cavity and the respiratory tract [6]. They are most often implicated in dental pathologies, such as caries, dental abscesses, and periodontal disease. They very rarely cause serious infections in immunocompetent individuals, but they can be opportunistic pathogens in immunocompromised hosts.* S. viridans* has been implicated in more serious diseases such as endocarditis, pneumonia, and bacteremia. This group of bacteria is a relatively rare cause of pyogenic liver abscess [2].

Foreign bodies can serve as an important nidus of infection as they allow bacteria to form biofilms [7]. *S. viridans* has this ability, which can allow for evasion of host immunity and protection against antibiotic therapy [3]. Because foreign body ingestions are most commonly found in pediatric populations, cases of foreign body ingestions in adults can be a diagnostic challenge and may not be attributed as a cause of systemic infection in adults. Furthermore, documented cases of foreign body ingestion are largely isolated to upper gastrointestinal discoveries and their complications such as dysphagia, cervical infection, and esophageal perforation. In this case, the foreign body traveled distally to his descending colon upon initial presentation. While 80 percent of ingested foreign bodies pass through the gastrointestinal tract without medical intervention, this case highlights the lack of treatment guidelines for foreign bodies in the distal gastrointestinal tract [8]. We further illustrate the complications resulting in not only primary pathologies, such as obstruction and perforation but also secondary pathologies such as bacterial abscess formation and sepsis.

In the presented case, our patient was fully immunocompetent with no risk factors or comorbidities that would increase the risk of forming PLA. He had no biliary or intra-abdominal pathology. Cultures did not grow microorganisms typically implicated in PLA, but, instead, they grew a commensal organism of the oral cavity that rarely causes abscess in healthy individuals. We believe the uniqueness of this case is attributed to a unique inciting event: the swallowing of a dental bridge. As the patient’s cultures grew *S. viridans*, we believe the swallowed dental bridge served as a nidus of infection and allowed for organisms of the oral cavity to form a biofilm, which was then able to cause systemic infection and abscess formation once the dental bridge was able to pierce the patient’s diverticula and become lodged in the descending colon. To our knowledge, no previous cases of *S. viridans *liver abscess secondary to a swallowed dental bridge have been described in the literature.

This case report highlights an interesting association between PLA and an unusual foreign body that was successfully treated with percutaneous drainage, intravenous antibiotics, and foreign body removal. From this case, we can also see the utility of broad-spectrum empiric antibiotic coverage as PLA are not always secondary to intra-abdominal pathology and microorganisms. It also shows the importance of early removal of foreign bodies before they have the opportunity to cause systemic infection.

## Conclusions

In conclusion, this case serves to highlight an interesting case of PLA secondary to a swallowed dental bridge. This case shows the importance of suspecting unusual pathogens and sources of infection as the most common sources of liver abscess (*E. coli*, *Enterococcus*) are not always the cause. We suggest starting with broad-spectrum antibiotic coverage when liver abscess is suspected and tailoring antibiotic treatment as the specific organism and susceptibilities are identified. We also suggest the importance of removing any foreign bodies promptly upon discovery as they may serve as important niduses of infection.
